# Controversies In The Surgical Management Of Shoulder Instability: Associated Soft Tissue Procedures

**DOI:** 10.2174/1874325001711010989

**Published:** 2017-08-31

**Authors:** Santos Moros Marco, José Luis Ávila Lafuente, Miguel Angel Ruiz Ibán, Jorge Diaz Heredia

**Affiliations:** Hospital MAZ - Orthopaedics and trauma surgery Avda Academia General Militar nº74, Zaragoza 50015, Spain

**Keywords:** Shoulder instability, Soft tissue, Shoulder dislocation, Instability copathology

## Abstract

**Background:**

The glenohumeral joint is a ball-and-socket joint that is inherently unstable and thus, susceptible to dislocation. The traditional and most common anatomic finding is the Bankart lesion (anterior-inferior capsule labral complex avulsion), but there is a wide variety of anatomic alterations that can cause shoulder instability or may be present as a concomitant injury or in combination, including bone loss (glenoid or humeral head), complex capsule-labral tears, rotator cuff tears, Kim´s lesions (injuries to the posterior-inferior labrum) and rotator interval pathology.

**Methods:**

A review of articles related to shoulder anatomy and soft tissue procedures that are performed during shoulder instability arthroscopic management was conducted by querying the Pubmed database and conclusions and controversies regarding this injury were exposed.

**Results:**

Due to the complex anatomy of the shoulder and the large range of movement of this joint, a wide variety of anatomic injuries and conditions can lead to shoulder instability, specially present in young population. Recognizing and treating all of them including Bankart repair, capsule-labral plicatures, SLAP repair, circumferential approach to pan-labral lesions, rotator interval closure, rotator cuff injuries and HAGL lesion repair is crucial to achieve the goal of a stable, full range of movement and not painful joint.

**Conclusion:**

Physicians must be familiarized with all the lesions involved in shoulder instability, and should be able to recognize and subsequently treat them to achieve the goal of a stable non-painful shoulder. Unrecognized or not treated lesions may result in recurrence of instability episodes and pain while overuse of some of the techniques previously described can lead to stiffness, thus the importance of an accurate diagnosis and treatment when facing a shoulder instability.

## 1 INTRODUCTION

The shoulder is the least constrained [1] and most mobile of all the joints in the human body and preservation of its stability is crucial to its function [2]. The bony architecture of the glenohumeral joint is often associated to a ball-and-socket geometry. This shape provides a large arc of motion, but also an inherent instability that can result in shoulder dislocations. In fact, the glenohumeral joint has been reported as the most frequently dislocated diarthrodial joint of the human body [3].

The glenohumeral joint stability relies on a complex combination of static and dynamic stabilizers [4] and compromise of these structures can lead to dislocation and often, recurrent instability.

The mechanisms and structures that confer static stability to the glenohumeral joint include bone architecture, negative intra-articular pressure, glenoid labrum, glenohumeral ligaments around the joint and coracohumeral ligament [5]. Dynamic stabilizers are rotator cuff, deltoid muscle, scapula-thoracic muscles and to a lesser extent, long head of the biceps tendón [6].

Due to the high number of anatomic structures that contribute to shoulder stability, the spectrum of injuries present in shoulder instability is broad [7-9]. Significant alterations appear during the first event of shoulder dislocation but additional injuries may occur with every recurrent dislocation episode [10].

The target for the physician should be to recognize all the injuries involved in shoulder instability and addressing them to become a stable, with full range of movement and not painful joint.

The aim of this article is to make a review of the soft tissue injuries that are present in shoulder instability, the procedures performed to address them during surgery and the controversies around this issue.

## 2 CAPSULO-LABRAL INJURIES AND PROCEDURES

The glenoid labrum deepens the glenoid cavity up to 50% [11], increases humeral contact, prevents rollback of the humeral head and represents the insertion line for capsule ligaments and long head of biceps [12]. Thus, injuries that cause detachment of the labrum from its anatomic insertion can originate some kind of shoulder instability.

**Fig. (1):**
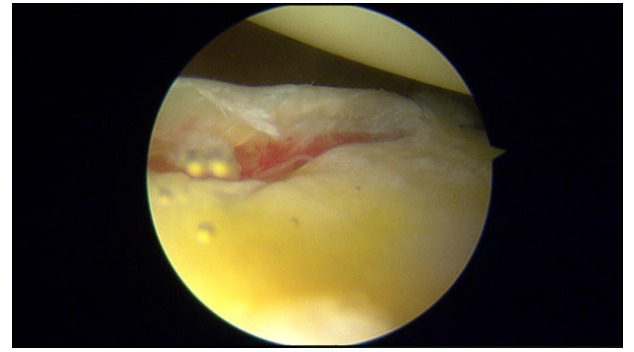
Bankart lesion in a right shoulder.

Bankart lesion, defined as the antero-inferior detachment of the glenoid labrum (Fig. **1**), is considered the “essential” lesion of anterior shoulder instability, however, it is not always present. It was found in 93.7% of the patients with anterior shoulder dislocation and it was more frequent in recurrent instability (78.2% of patients with acute instability and 97.11% of patients with chronic instability) [10]. According to Werner *et al.* [8], in patients with atraumatic shoulder instability that do not respond to a rehabilitation program, physicians must suspect intra-articular injuries similar to those found in post-traumatic subluxations or dislocations, although in a lower percentage. Thus, Bankart lesion can also be found in symptomatic atraumatic shoulder instability in the context of a pathologic capsular laxity. Besides that, in experimental studies that recreated instability conditions, Bankart lesion was not enough by itself to cause shoulder dislocation; superior and posterior extension is needed before the external rotation and abduction traumatic mechanism which caused dislocation [13].

Although Bankart repair is not a direct objective of this review, as a controvertial issue, regarding Bankart lesion treatment, we would like to mention that Ozturk *et al.*, found that mattress repair was associated with a higher rate of return to sports at the pre-injury level compared to those who underwent a stabilization by simple knots (89% of the patients who underwent a labral repair with a mattress configuration returned to pre-injury sport level while only 62% of those who underwent a stabilization with simple knots recovered that level with no significant differences regarding the rate of recurrence) [14].

Along the following text, we will present the controversies surrounding the management of the most frequent labral and capsulo-labral injuries and procedures performed for their management during shoulder instability surgery.

## 3 SLAP TEARS

Injuries to the superior glenoid labrum are identified as an important source of shoulder pain and dysfunction. These injuries can present either isolated or in association with a wide spectrum of shoulder pathological conditions including instability, illustrating the challenge in the diagnosis of SLAP tears and instability.

According to Snyder classification into 4 distinct types [15] and the posterior modifications by Maffet *et al.* [16] (adding 3 more types), Morgan and colleagues [17] (expanding type II into three different injury patterns) and Powell *et al.* [18] (adding 3 more types of SLAP lesions to the classification), according to pathoanatomy, we can understand that most of these subtypes of SLAP tears are themselves a clear cause of shoulder instability.

The prevalence of SLAP lesions was not different between patients with acute instability and chronic cases in Yiannakopoulos series (21.73% of acute and 20.19% of chronic instabilities presented a SLAP tear), but the more severe subtypes of SLAP lesions present in this series (III-IV) appeared in patients with chronic instability, possibly as the result of the recurrent episodes of dislocation [10].

**Fig. (2):**
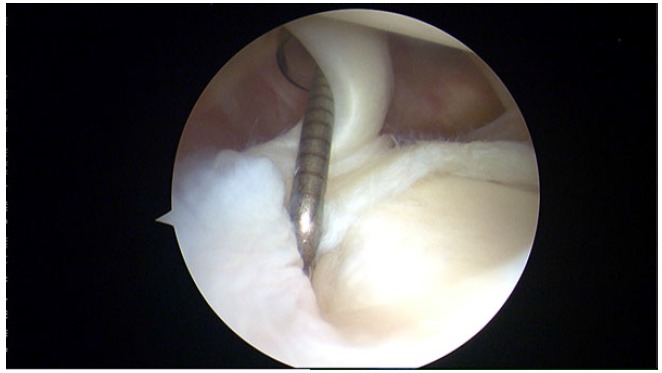
SLAP type II lesion in a right shoulder.

SLAP type II lesions (Fig. **2**) which are most commonly seen in daily practice and there has been some controversies about its treatment. However, some authors advocate for biceps tenodesis [18], others support the labrum repair based on good outcomes even in patients older than 40 years old [19].

 Despite this controversy, there is one article that demonstrates an increasing number of patients undergoing arthroscopic SLAP repair in the United States [20]. Regarding the surgical technique for repairing the SLAP type II, most surgeons recommend to address the labrum zone posterior to the biceps tendon only, in order to avoid excessive tightening of the middle glenohumeral ligament that may lead to loss of external rotation [21], while others fix both regions around the biceps tendon (11 and 1 o´clock) taking care not to cause torsion of this structure [18].

**Fig. (3):**
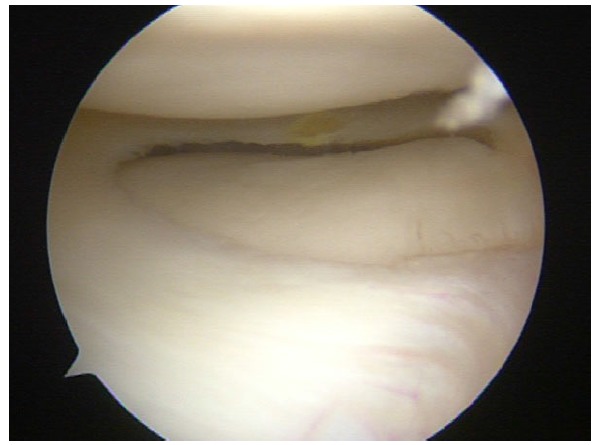
Panlabral injury in a right shoulder.

For the rest of SLAP tears, including combinations of SLAP II with other capsule-labral injuries and pan-labral injuries (Fig. **3**), most authors strongly recommend a circumferential approach to capsule and labrum repair depending on the patient´s complaints and physical examination at clinics and under anesthesia. Special attention is mandatory in patients who require both, labrum repair and capsule plication, in order to fix all the lesions adequately [9]. In this recent review [9], the use of a minimum of 3 anchors below the midline of the glenoid (3 o´clock for a right shoulder) is recommended to get a good capsule-labral plicature as part of the global approach to the shoulder injury.

In relation to the sequence of a pan-labral repair or multi-injured labrum, some authors recommend to start fixing post-inferior labrum, then inferior and anterior labrum (5 and 7 portals may be useful), and the last one is the SLAP lesion between 11 and 1 o´clock positions in order to optimize arthroscopic visualization [9, 22], but others perform the anatomic fixation of the superior labrum first, in order to establish an anatomical reference, and then fix the rest of the labrum as previously described [22].

## 4 ALPSA

**Fig. (4):**
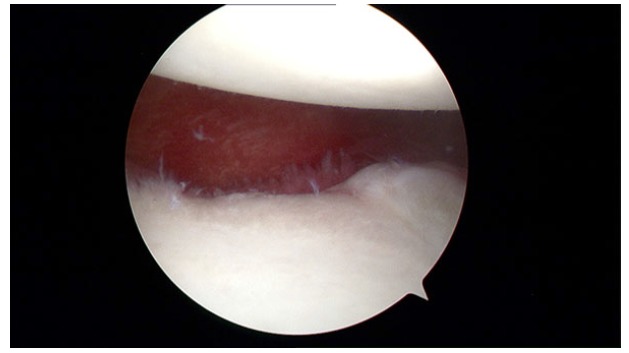
ALPSA lesion in a right shoulder.

First reported by Neviaser, it is the acronym for Anterior Labroligamentous Periosteal Sleeve Avulsion, lesion where the torn labrum and IGHL (Inferior GlenoHumeral Ligament) have healed more medially on the anterior aspect of the glenoid neck (Fig. **4**) [23]. Some studies revealed that it is more common among younger patients (< 25a) and it is associated with a higher number of preoperative dislocation events [24, 25]. According to Yiannakopoulos series, no ALPSA lesion was found in patients with acute instability and 100% of ALPSA lesions were noted in patients with chronic instability (13 out of 104 patients with recurrent instability) [10].

When repairing an ALPSA lesion, it is essential to identify and completely liberate the periosteum layer in order to mobilize the labrum and repair it in the anatomic footprint. Double-row suture anchor techniques, with a similar concept to those used in rotator cuff repair, have been described to recreate the native anatomy and to increase the contact area between capsule and glenoid bone, therefore decreasing the high dislocation recurrence rate of these injuries [26, 27]. These techniques showed good results in terms of restoring the native footprint better than single row techniques, but still have not been widespread.

## 5 KIMS'S LESION

This injury, described by Kim *et al.* in 2004 consists of a superficial tear between posteroinferior labrum and glenoid cartilage without complete detachment of the labrum, including lost of normal height of the labrum and retroversion of the chondrolabral glenoid [28]. It is typically present at the 6 to 9 o´clock position for the right shoulder and when probing it, fluctuation and loose attachment of the labrum is noted.

**Fig. (5):**
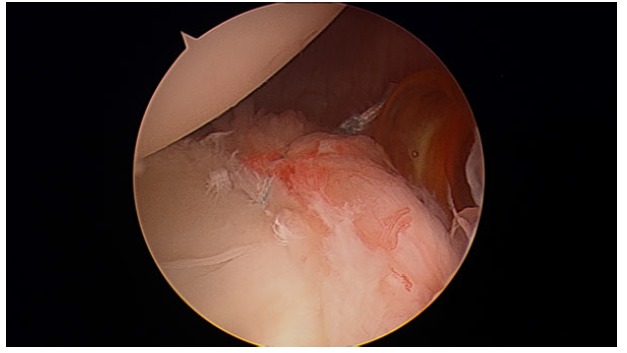
Repaired Kim´s lesion in a right shoulder.

Treatment includes labroplasty and capsular shift as the injury is often a part of posterior or multidirectional instability. Three portals are needed: 2 anterior and 1 posterior portal 1cm lateral to the standard soft spot posterior portal. This posterior portal provides an ideal access to posteroinferior labrum and capsule. Viewing from the anterior superior portal, a tissue liberator is introduced through posterior portal and the superficial part of the chondro-labral junction is incised, thus viewing the detachment of the deep portion of the labrum. After bone and soft tissue refreshing, the capsule-labral shift is performed (Fig. **5**).

## 6 POSTEROINFERIOR CAPSULE PLICATION

**Fig. (6):**
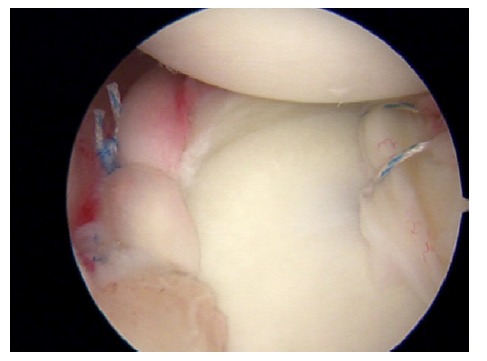
Bankart repair plus posteroinferior capsule plication in a right shoulder.

This procedure is performed to reduce posterior capsular volume and address capsular redundancy for treatment of posterior instability but it is also commonly used to increase stabilization of an anterior capsule-labral repair or in patients with evident anterior instability without labral tear (Fig. **6**) [29].

Capsular plication must be performed adequately and according to the patient´s needs. Less plication than required results in capsular redundancy and overtightening of the capsule may result in loss of range of movement [30]. Repair techniques with and without anchors have been used. Plicature with anchors is the preferred technique when there is damage to the labrum [31]. Capsule plication using stitches only, where sutures pass through the margin between labrum and glenoid cartilage, has shown good results concluding that intact labrum offers similar fixation strength to a glenoid anchor but notifying that labrum displacement up to 1.5mm may be expected without the use of anchors [32]. Therefore, it is crucial to search for any evidence of labral injury while operating, as plication with suture anchors might provide a more secure repair in this group of patients [30]. When labrum is intact and the plication is made without anchors, simple, mattress and figure of eight stitches can be used effectively, but simple stitch is easier and may cause less trauma to the capsulolabral complex [30].

## 7 ROTATOR INTERVAL CLOSURE

The rotator interval is a part of capsular tissue between the superior aspect of the subscapularis and the anterior border of the supraspinatus tendons composed by superior glenohumeral ligament (SGHL), glenohumeral capsule and coracohumeral ligament. The rotator interval functions are: (1) Resist inferior translation of the humeral head in the adducted shoulder, (2) resist posterior translation of the humeral head with shoulder in external rotation and flexion or adduction and (3) limit the external rotation [33]. Thus, rotator interval pathology is recognized as an important factor in shoulder instability [34] and its closure is an additional option for unstable shoulders. The present rotator interval closure concept is simple: plicating the superior and inferior borders of this structure with sutures in order to decrease capsular volume or address rotator interval laxity. The main question regarding rotator interval closure is when to perform it because the indications still remain poorly defined [35]. This technique is not recommended as a routine practice in shoulder multidirectional instability, isolated posterior or inferior instability as the results are controversial. In specific patients, rotator interval closure may help to address anterior instability by decreasing capsular volume and reducing laxity but this is often achieved at the cost of postoperative loss of external rotation [9]. Therefore, It should be performed tailored to the patients needs, typically when “sulcus test”, that is used to assess the integrity of the rotator interval, is positive (subluxation of the humeral head in zero degrees of abduction and in external rotation) [6]. Other authors recommend to repair rotator interval after arthroscopic shoulder instability surgery because this area has been weakened by the placement of two cannulas and can be origin to recurrent instability [36, 37]. Also, isolated rotator interval closure has been advocated in patients with mild inferior laxity with positive sulcus test but no radiographic or magnetic resonance imaging evidence of labral or capsular tears [38]. Several techniques have been described to close the rotator interval. One of the most commonly used techniques consists in passing two nº1 sutures through SGHL and MGHL helped by cannulas that are backed out of the glenohumeral joint just anterior to the capsule. After the sutures have been passed through, the surgeon has to tie them by a blind knot through one of the cannulas plicating the rotator interval.^35^ Regardless the chosen technique, it is crucial to externally rotate the shoulder in some degrees to avoid overtightening of the closure leading postoperative loss of external rotation [35, 38]. According to Randelli *et al.* results in a homogenous series of patients [39], the average loss of external rotation after Bankart repair with rotator interval closure associated was 12.14 degrees along the side of the arm and 7.21 degrees at 90º of flexion what represents 17´8% and 8% of the arc of motion,getting 85.7% of good to excellent results in Rowe Score. The authors´opinion about rotator interval closure is that although it seems a simple procedure, it is not and a refined technique should be employed to avoid complications by dramatically decreasing external rotation.

## 8 ROTATOR CUFF TEARS AND INSTABILITY

**Fig. (7):**
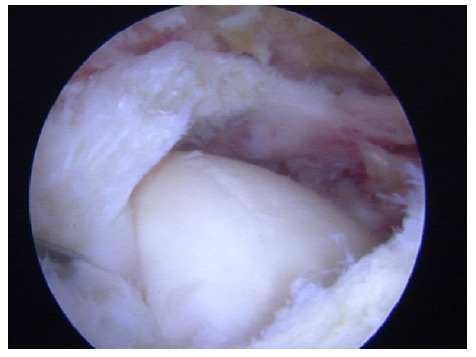
Supraspinatus and infraspinatus tear in a right shoulder after first episode of instability.

The rotator cuff is considered to have an important role in static and dynamic stability of the glenohumeral joint as it creates a concavity-compression mechanism resulting in restraint to the joint [40, 41]. A rotator cuff tear is more probable a consecuence of a shoulder dislocation rather than the cause, but both situations can happen due to the shoulder mechanics [42]. In fact, in a cadaveric study, a 50% of decrease in rotator cuff activity turned in an increase of 50% of dislocations and also a smaller capsule-labral tear was needed to provocate instability in the presence of a cuff defficience [43]. The frequency of rotator cuff tears after an episode of anterior dislocation varies between 7 and 32% and increases as the age and number of dislocations do [44, 45]. A higher number of dislocations is strongly correlated with a higher number of posterosuperior injuries to the rotator cuff [45] while subscapularis tears are associated with acute severe posterior or anterior instability events [9]. Physicians should suspect concomitant injury to the rotator cuff in patients older than 40 years with persistent pain and weakness two to three weeks after an anterior dislocation, and an MRI should be performed (Fig. **7**) [44]. This persistent dysfunction was often attributed to a nerve palsy, resulting in delayed diagnosis of the rotator cuff tear, but the three lesions can be present in what is called the “terrible triad of the shoulder” [46].

Once the diagnosis has been established, treatment options must be considered. In some patients with a balanced cuff lesion, minimal shoulder pain and without instability, nonoperative treatment can be a good option [47]. According to a recent review [44], the rest of the patients with concomitant shoulder instability and rotator cuff injuries, may benefit from shoulder stabilization and rotator cuff repair despite the postoperative risk of shoulder stiffness. In relation to the timing of surgery, Lahteenmaki *et al.* [48] reported better results after early treatment (24 of 26 patients, 92%, with good or excellent results according to UCLA shoulder rating scale), taking into account that as time passes by, the tear may enlarge, and the cuff may lose elasticity, making the late surgical repair more difficult or even impossible, thus the importance of a prompt diagnosis.

When a SLAP tear (as the instability main mechanism) and a rotator cuff tear are the concomitant injuries, also a potential risk of postoperative stiffness has been described [21], however, several authors have reported complete range of motion after both injuries repair in the same procedure [49, 50].

## 9 HAGL LESIONS

**Fig. (8):**
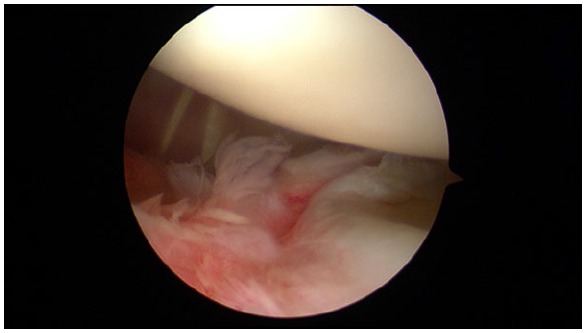
HAGL lesion in a right shoulder. View from anterosuperior portal.

Humeral avulsion of the glenohumeral ligaments (HAGL) is a recognized cause of recurrent shoulder instability. Firstly described by Nicola [51], it was not until 1995 when Wolf *et al.* [52], referred to this injury as HAGL or humeral avulsion of the glenohumeral ligaments (Fig. **8**). HAGL lesions typically occur by trauma with hyperabduction and external rotation of the arm resulting in incompetence of the IGHL complex thus leading to glenohumeral instability, reproduced mainly in abduction and external rotation. This injury has been reported in 2% to 9% of patients with shoulder instability [51, 53, 54].

 According to Bui-Mansfield *et al.*[54] this lesion can be classified into 6 different types based on anterior or posterior affection, presence or absence of bony detachment and association or not with labral injury. Anterior HAGL forms represent about 93% of the cases while posterior HAGL are only the 7% [53]. A recent study, based on a cadaver model, reports no alteration of shoulder kinematics with small HAGL lesions (18.4 ± 1.8 mm) compared to a normal shoulder, whereas large HAGL lesions (36.8 ± 3.6 mm) increase ROM and glenohumeral translation in the scapular and coronal plane, conditions that are restored after HAGL lesion repair [55]. Regarding diagnosis, MRI is the best option to address a HAGL lesion when it is suspected and it is best visualized on coronal oblique or sagittal oblique T2-weighted fat-suppressed images [56]. These injuries seldom appear isolated and concomitant capsule-labral injuries should be suspected when glenohumeral instability is developed after trauma. In fact, underdiagnosed HAGL lesions can be a cause of recurrent instability after primary stabilization [57]. Surgical treatment is indicated for young athletes and manual laborers and for those patients with persistent pain and instability after a rehabilitation program. Arthroscopic repair can be performed, more easily in lateral decubitus to facilitate access to inferior capsule, particularly by using accessory advanced portals (5 portal, 7 portal and Bathia portal) [58, 59]. Placing the arm in abduction and external rotation to reach the humeral neck, bone is debrided, suture anchors are placed and horizontal mattress sutures are passed through the glenohumeral ligament to reduce the tissue to the footprint avoiding to overtight the capsule. Although most reports are small case series, results of arthroscopic management are promising [60].

## CONCLUSION

Due to the complex anatomy of the shoulder and the large range of movement of this joint, a wide variety of anatomic injuries and conditions can lead to shoulder instability, specially present in young population. Physicians must be familiarized with all the lesions, and should be able to recognize and subsequently treat them to achieve the goal of a stable non-painful shoulder. Unrecognized or not treated lesions may result in recurrence of instability episodes and pain, while overuse of some of the techniques previously described can lead to stiffness, thus the importance of an accurate diagnosis and treatment is essential when facing a shoulder instability.
